# Synergistic effect of therapeutic stem cells expressing cytosine deaminase and interferon-beta via apoptotic pathway in the metastatic mouse model of breast cancer

**DOI:** 10.18632/oncotarget.6719

**Published:** 2015-12-22

**Authors:** Bo-Rim Yi, Seung U. Kim, Kyung-Chul Choi

**Affiliations:** ^1^ Laboratory of Biochemistry and Immunology, College of Veterinary Medicine, Chungbuk National University, Cheongju, Chungbuk, Republic of Korea; ^2^ Department of Medicine, Faculty of Medicine, University of British Columbia, Vancouver, British Columbia, Canada; ^3^ TheraCell Bio and Science, Cheongju, Chungbuk, Republic of Korea

**Keywords:** breast cancer, metastasis, interferon-beta, 5-fluorocytosine, stem cell therapy

## Abstract

As an approach to improve treatment of breast cancer metastasis to the brain, we employed genetically engineered stem cells (GESTECs, HB1.F3 cells) consisting of neural stem cells (NSCs) expressing cytosine deaminase and the interferon-beta genes, HB1.F3.CD and HB1.F3.CD.IFN-β. In this model, MDA-MB-231/Luc breast cancer cells were implanted in the right hemisphere of the mouse brain, while pre-stained GESTECs with redfluorescence were implanted in the contralateral brain. Two days after stem cells injection, 5-fluorocytosine (5-FC) was administrated via intraperitoneal injection. Histological analysis of extracted brain confirmed the therapeutic efficacy of GESTECs in the presence of 5-FC based on reductions in density and aggressive tendency of breast cancer cells, as well as pyknosis, karyorrhexis, and karyolysis relative to a negative control. Additionally, expression of PCNA decreased in the stem cells treated group. Treatment of breast cancer cells with 5-fluorouracil (5-FU) increased the expression of pro-apoptotic and anti-proliferative factor, BAX and p21 protein through phosphorylation of p53 and p38. Moreover, analysis of stem cell migratory ability revealed that MDA-MB-231 cells endogenously secreted VEGF, and stem cells expressed their receptor (VEGFR2). To confirm the role of VEGF/VEGFR2 signaling in tumor tropism of stem cells, samples were treated with the VEGFR2 inhibitor, KRN633. The number of migrated stem cells decreased significantly in response to KRN633 due to Erk1/2 activation and PI3K/Akt inhibition. Taken together, these results indicate that treatment with GESTECs, particularly HB1.F3.CD.IFN-β co-expressing CD.IFN-β, may be a useful strategy for treating breast cancer metastasis to the brain in the presence of a prodrug.

## INTRODUCTION

Metastasis is the movement of cancer cells from primary tumor sites to distant organs and tissues, including the brain, liver, and bones, via the blood and lymphatic vessels [1]. Metastasis is the result of several sequential steps, intravasation, invasion, extravasation, and micrometastases [2, 3]. Lung (40–50%), breast (15–25%), melanoma (5–20%), colon and kidney related primary cancer commonly spread to the brain [4]. About 80% of metastatic lesions are located in the cerebrum hemispheres, while 15% are located in the cerebellum and 5% in the brainstem [5]. Depending on the location of the brain metastases, patients may suffer from neurological symptoms that include headaches, focal weakness, mental disturbance, seizures, and ataxia [6]. Metastasis is responsible for most cancer deaths [7]. Breast cancer metastases are the second most common type of brain metastases, followed by lung cancer, and these are generally found in younger and premenopausal women [8]. Breast cancer metastases are also more common in women with triple negative or human epidermal growth factor receptor 2 (HER2)/neu positive breast cancer [9]. For breast cancer patients, the prevalence of brain metastases has historically been estimated to be 10–16% with a 1-year survival rate of 20% [10]. Therapeutic approaches to brain metastases include chemotherapy, surgery, whole brain radiotherapy (WBRT) and stereotactic radiosurgery (SRS) supplemented with corticosteroid therapy for symptomatic relief [11]. While surgical treatment of the primary tumor may be successful, therapies for metastatic breast cancer carry the risk of neurological and cognitive deficits. Recently, treatment of MDA-MB-231, aggressive breast cancer cells, with a monoclonal antibody specific for ROR1 inhibited cancer cell migration and invasion *in vitro* and tumor metastasis *in vivo*, indicating that ROR1 may suppress breast cancer progression and metastasis via regulating epithelial-mesenchymal transition (EMT) [12].

As an alternative therapy for metastatic breast cancer, neural stem cells (NSCs) derived from human fetal telencephalon tissues at 15 weeks were used for neural stem cell-directed enzyme/prodrug therapy (NDEPT) [13, 14]. The NSCs were immortalized using a retroviral vector carrying v-myc oncogene and were genetically engineered to express therapeutic genes, bacterial cytosine deaminase (CD) and human interferon-beta (IFN-β) [15]. The CD gene, which is a suicide genes, can convert 5-fluorocytosine (5-FC), a non-toxic agent, into 5-fluorouracil (5-FU), a toxic agent, which inhibits DNA synthesis and induces apoptosis in cancer cells [16]. Additionally, IFN-β is a member of the type I IFN family (IFN-alpha (IFN-α) and IFN-omega (IFN-ω)) that suppresses tumor cell growth via the induction of differentiation, S-phase accumulation and apoptosis [17]. Although high concentrations of IFN-β are known to inhibit malignant cell growth *in vitro*, the *in vivo* therapeutic utility is limited by its excessive toxicity when administered at high doses [18]. Because it has shown limited response owing to its short half-life, IFN-β cannot reach the concentration required to suppress tumor cell growth [19]. The results of several *in vitro* and *in vivo* investigations of the treatment of hepatocellular carcinoma (HCC) showed that modulation of the tumor necrosis factor-related apoptosis-including ligand (TRAIL)/TRAIL receptor-mediated cytotoxic pathway might partially contribute to the clinical efficiency of combined treatment with 5-FU and IFN-β without hepatotoxicity or toxicity against normal tissues [20]. In the previous study, type I IFN receptor type 2 (IFNAR2)-positive cancer cells showed a positive clinical response to combination therapy of type I IFNs and 5-FU [21]. Wada *et al.* also confirmed that synergistic and anti-angiogenic effects of IFN-α and 5-FU combination therapy may contribute to the anti-tumor effects against HCC through regulation of vascular endothelial growth factor (VEGF) and angiopoietins [22].

Stem cells can also migrate toward tumors to interact with several growth factors secreted by tumor cells [23]. The therapeutic treatment by engineered stem cells is a novel strategy where the combination of the migration capacity of stem cells as a vector for therapeutic genes towards diverse human tumors [13, 14, 16, 17, 24–26]. A synergistic antitumor effect of CD and IFN-β genes can selectively target these types of human cancers [27]. However, the molecular mechanisms for NSCs mobilization against various tumors have not been identified. In another study, exposure of stromal cell-derived factor 1 alpha (SDF-1α) to quiescent NSCs was found to enhance proliferation, promote chain migration and transmigration, and activate intracellular molecular pathways mediating engagement [28]. Schmidt *et al.* demonstrated that VEGF is a strong signal for guiding the *in vivo* migration of NSCs from distant sites in the adult brain [29]. VEGF is a typical angiogenic growth factor that acts as a potent mitogen and is known for exerting neuroprotective effects against ischemic injury [30]. Therefore, undetectable dormant single metastatic cells or prevascular micrometastases can be treated via tumor tropic properties of stem cells expressing therapeutic genes.

The present study describes the potential for use of genetically engineered stem cells (GESTECs) to reduce tumor growth via tumor tropic effects in metastatic breast cancer animal models. Our results showed that synergistic effects of 5-FU and IFN-β co-treatment in a breast cancer cell line can lead to apoptosis and proliferation related protein activation through p53 and p38. We also investigated whether NSCs have a significant capacity to migrate via VEGF signaling as well as a therapeutic effect. Overall, the results of this study demonstrate the potential for use of NDEPT-based IFN-β therapy to treat breast cancer metastasis to the brain.

## RESULTS

### Metastatic breast cancer mice models and therapeutic effects of stem cells

**Figure 1:**
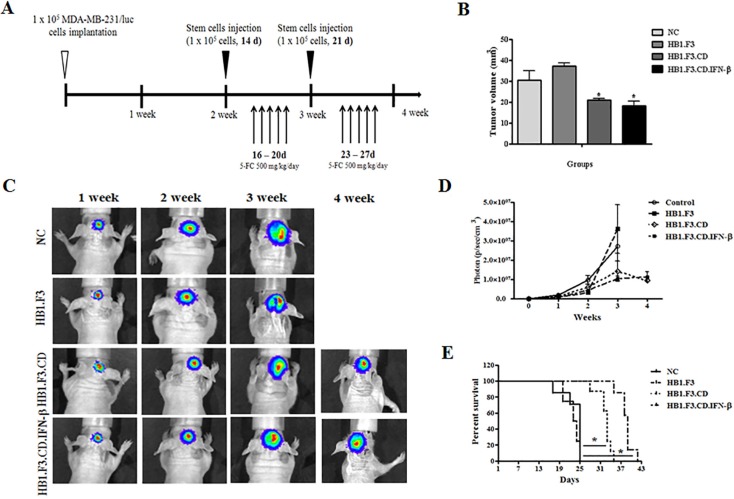
Mouse model for breast cancer metastasis to brain and therapeutic effect of stem cells expressing therapeutic genes, cytosine deaminase (CD) and/or interferon-beta (IFN-β) Luciferase labeled MDA-MB-231 breast cancer cells were implanted in the right hemisphere of anesthetized nude mice (AP + 1.0 mm, ML + 1.7 mm, DV −3.2 mm). Three stem cell lines, HB1.F3, HB1.F3.CD, and HB1.F3.CD. IFN-β cells, were transplanted to the opposite hemisphere of mice after two weeks. To activate the CD gene in stem cells, 5-FC (500 mg/kg/day) was administrated as a prodrug via intraperitoneal (*i.p.*) injection once a day 5 days per week for 2 weeks. (**A**) Schematic diagram of mouse models. (**B**) Volume of excised brain tumor. After the final 5-FC injection, brains were excised from several mice to measure tumor burden inside the brain (*n* = 8). (**C**) Bioluminescence imaging. During the experimental period, living images of all groups of mice were acquired at the indicated weeks. (**D**) Bioluminescence values (photons/sec/cm^2^/sr) (*n* = 8). (**E**) Survival rate of mouse models. Data shown are the mean ± SEM. *p < 0.05 *vs*. negative control (*n* = 8).

As shown in Figure 1A, we demonstrated the therapeutic effect of hNSCs with 5-FC in breast cancer metastasis to the brain. To monitor the effects of hNSCs on the growth and metastasis of MDA-MB-231/Luc cells in live mice, cells were implanted into the mouse brain. In this experiment, one week after MDA-MB-231/Luc cells implantation, 32 mice were randomly divided into four groups. Three groups of mice received stem cells (HB1.F3, HB1.F3.CD, HB1.F3.CD.IFN-β cells) and 5-FC (500 mg/kg/day) treatment, while another group of mice received only the vehicle (saline). The antitumor effect of hNSCs expressing therapeutic genes with 5-FC was evaluated weekly after injection of stem cells by measurement using an IVIS imaging system (Figure 1C). The bioluminescence imaging results indicated a significant decrease in the tumor volume of HB1.F3.CD or HB1.F3.CD.IFN-β cells with 5-FC treated mice relative to negative control or HB1.F3 cells treated mice at 4 weeks (Figure 1D). At four weeks, we euthanized several mice, excised their xenograft breast cancer and measured the volume using a veterinary caliper. Significant inhibition of tumor growth (approximately 30–40%) was observed relative to the negative control or HB1.F3 cells treated groups in HB1.F3.CD or HB1.F3.CD.IFN-β cell treated groups (Figure 1B). These results were in accordance with the results of bioluminescence imaging of live mice. Moreover, all mice in the negative control or HB1.F3 cells injected groups survived until day 25. However, HB1.F3.CD.IFN-β and 5-FC co-treated mice survived significantly longer than any other groups, living until day 43 (Figure 1E). In the HB1.F3.CD plus 5-FC treated group, all mice were still alive at day 35. It should be noted that mice treated with HB1.F3.CD or HB1.F3.CD.IFN-β in the presence of 5-FC showed a prolonged survival rate when compared with the untreated control or HB1.F3 treated groups.

### Histopathological analysis of brain sections

**Figure 2:**
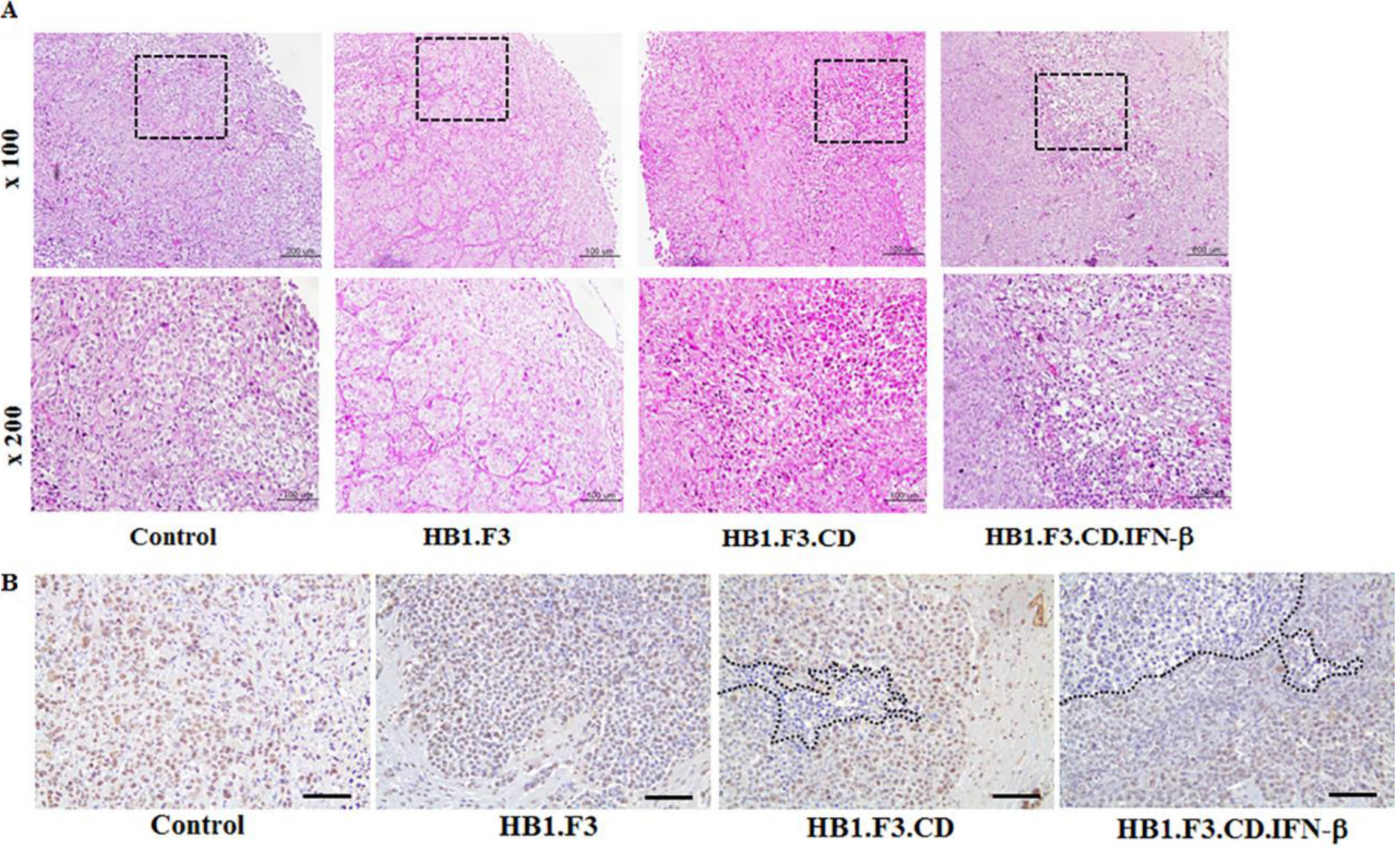
Hematoxylin and eosin (H & E) staining and PCNA expression level in metastatic breast cancer models Excised brains were fixed in 4% normal formalin and embedded in paraffin. After being cut with a microtome, slides were deparaffined and rehydrated with xylene, ethanol, and tap water. (**A**) H & E staining. (**B**) Immunohistochemistry (IHC) staining for PCNA. To confirm the proliferation rate of tumor cells, brain sections were treated with the primary antibody, anti-mouse PCNA. Next, the slide was incubated with biotinylated anti-mouse secondary antibody and stained protein (brown color) was observed following DAB and hematoxylin staining. Dotted line: necrosis or apoptosis area of tumor cells in the brain section. Magnification ×100, ×200.

We conducted histopathological analysis of excised metastatic breast cancer in all groups of mice. Histopathological examination of excised brains of mice from all groups confirmed the growth of breast cancer in the mouse brain. H & E staining showed that cancer cells grew well in the negative control or HB1.F3 cells treated groups, with clear atypia and no major tumor necrosis (Figure 2A). Conversely, HB1.F3.CD cells in the 5-FC treated group showed smaller zones of cancer cell necrosis or apoptosis. Progression of tumor necrosis or apoptosis occurred more frequently in HB1.F3.CD.IFN-β cells plus 5-FC treated mice. In the treatment with HB1.F3.CD or HB1.F3.CD.IFN-β cells, the aggressiveness of cancer cells, nuclear size or density were significantly decreased.

IHC staining for PCNA in metastatic breast cancer sections of all mice was used to investigate the overall pattern of expression. Proliferative tumor cells showed stronger nuclear staining in negative control or HB1.F3 treated mice than in HB1.F3.CD or HB1.F3.CD.IFN-β plus 5-FC treated mice (Figure 2B). In groups treated with stem cells expressing CD and/or IFN-β genes, expression of the PCNA protein decreased significantly in tumor burden of the brain, with a greater reduction occurring in the HB1.F3.CD.IFN-β cells treated group.

### Inhibition of tumor tropic effect of stem cells via VEGF/VEGFR2 signaling

**Figure 3:**
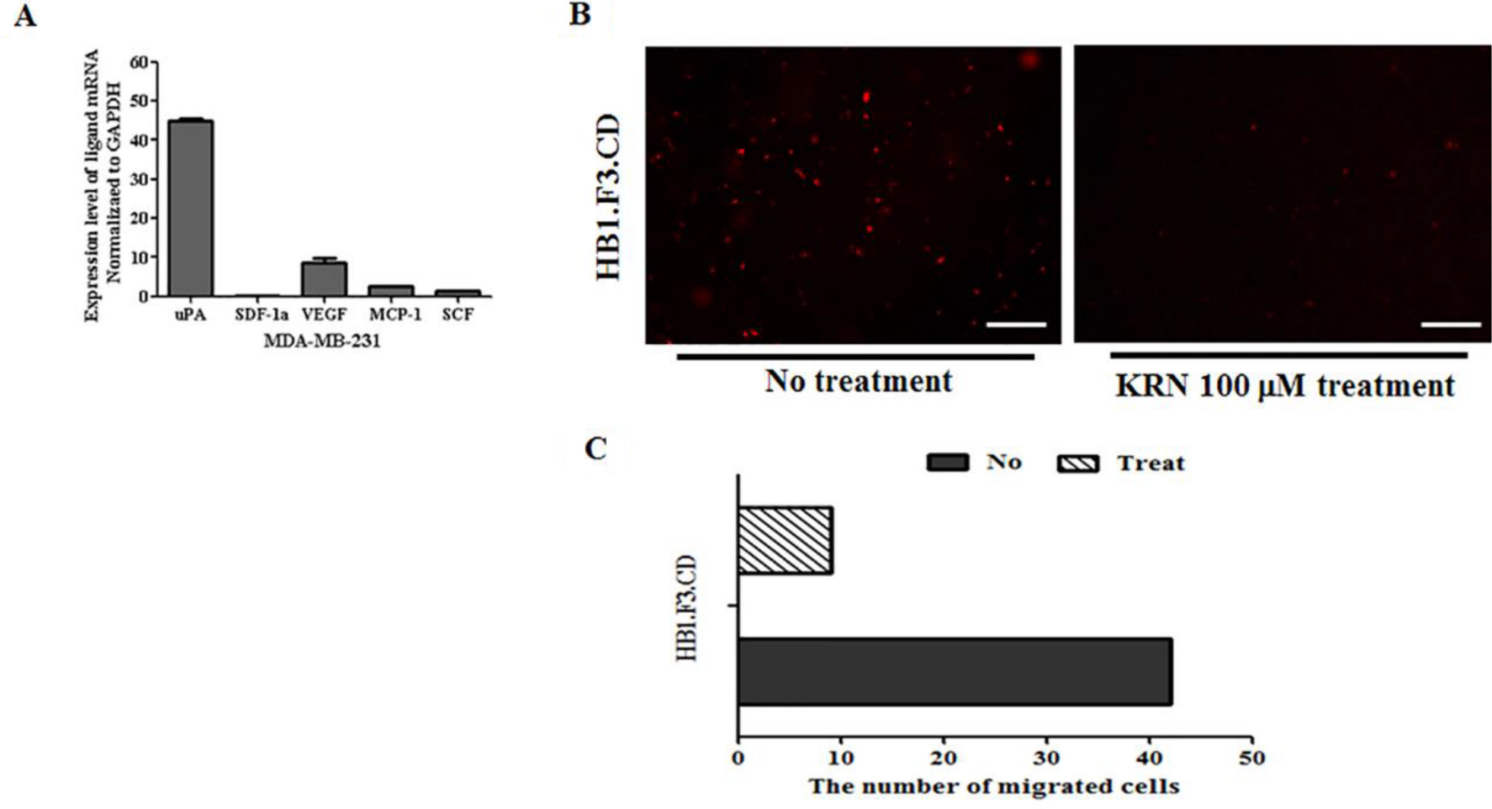
Effects of the vascular endothelial growth factor and their receptor 2 (VEGF/VEGFR2) pathway on stem cells migration (**A**) Chemoattractant factor secreted by MDA-MB-231 breast cancer cells. Total RNA was obtained from MDA-MB-231 and investigated by real time PCR with SYBR green and ROX dye. (**B**) Transwell assay after treatment with the VEGFR2 inhibitor, KRN633. After treatment of stem cells in the culture dish with 100 μM KRN633, CM-DiI stained HB1.F3.CD cells were seeded in the upper chamber of the transwell for 1 day. The next day, non-migrated cells were removed from the upper chamber of the transwell and the number of migrated cells was counted by fluorescent microscopy. (**C**) The number of migrated and non-migrated cells following KRN633 treatment. Magnification ×100.

The migration of hNSCs toward MDA-MB-231/Luc was assessed using transwell chambers. Migration ability of stem cells was dramatically influenced by several chemoattractant factors (uPA, VEGF, MCP-1, and SCF) secreted by MDA-MB-231/Luc cells (Figure 3A). To confirm these results, we conducted an inhibition study using KRN633 as a VEGFR2 inhibitor. HB1.F3.CD cells were pre-treated with the inhibitor (100 μM) for 1 h before the migration assay. As shown in Figure 3B, the number of migrated CM-DiI labeled hNSCs (stained in red) was significantly lower in the KRN633 pre-treated group than the KRN633 non-treated group. Overall, the amount of stem cells that migrated toward breast cancer cells was four times lower in the VEGFR2 signaling inhibited group (Figure 3C).

### Downregulation of VEGF/VEGFR2 signaling following KRN633 treatment

**Figure 4:**
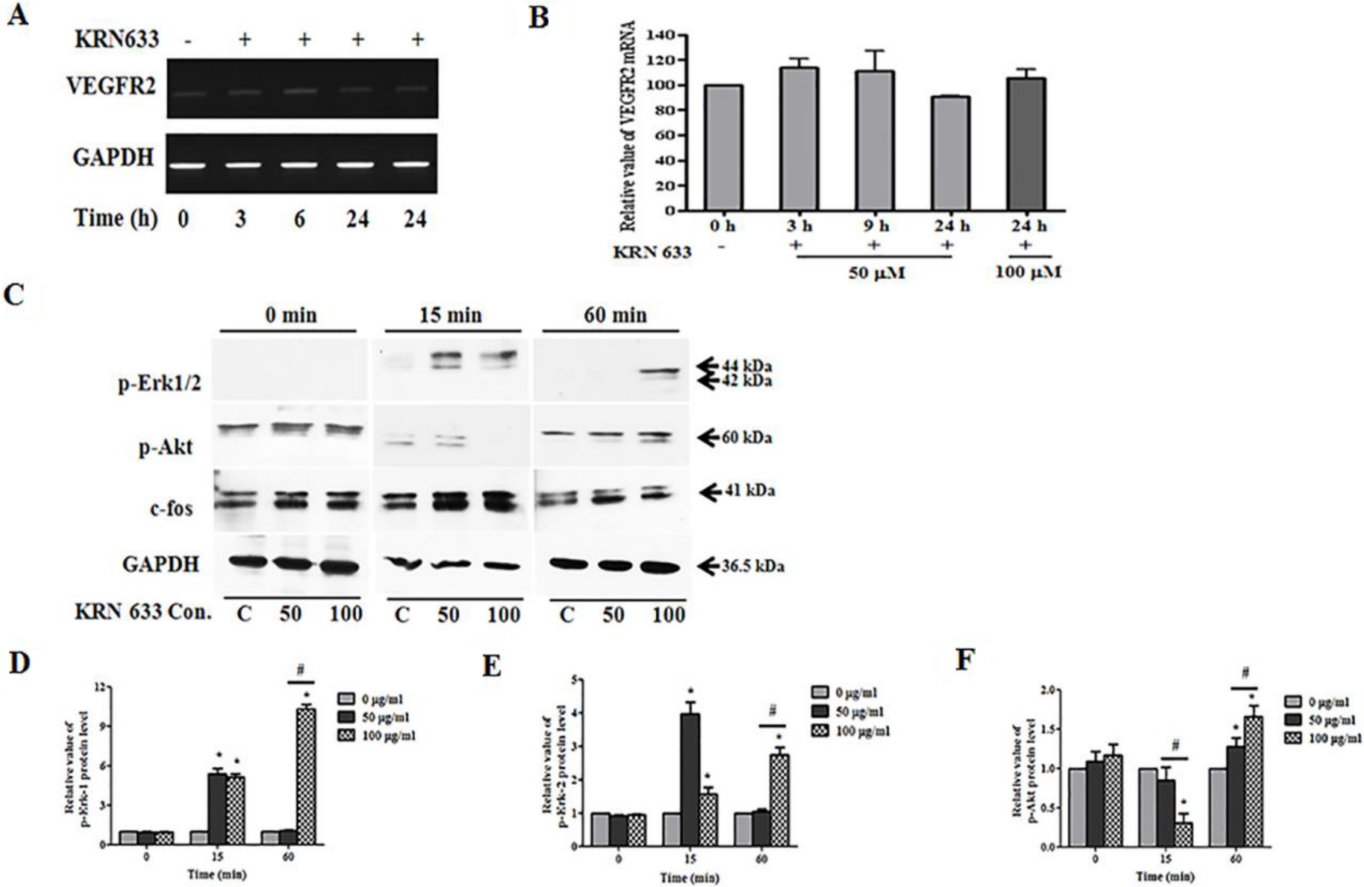
Alteration of VEGF/VEGFR2 signaling-related RNA and protein expression in stem cells following treatment with the vascular endothelial growth factor receptor 2 (VEGFR2) inhibitor, KRN633 (**A**) Expression of VEGFR2 gene in HB1.F3 cells. KRN633 at 50 μM or 100 μM was applied to HB1.F3 cells for up to 24 hours, during which time RNA was extracted at 3, 6, 9, and 24 h. To confirm the effects of KRN633, expression of the VEGFR2 gene was analyzed by RT-PCR. (**B**) Graph of the relative VEGFR2 mRNA level. (**C**) Expression of phospho-Erk1/2, phospho-Akt1/2/3, and c-fos protein in the HB1.F3.CD. Following KRN633 treatment (50 μM or 100 μM), protein was collected and separated by SDS-PAGE, then transferred to PVDF membrane and incubated with primary antibody, anti-p-Erk1/2, anti-p-Akt1/2/3, and anti-c-fos. (**D**) Graph of p-Erk1 related values. (**E**) Graph of p-Erk2 related values. (**E**) Graph of p-Akt1/2/3 related values. Each experiment was performed in triplicate and data shown are the mean ± SD. C: negative control (no treatment with KRN633) *p < 0.05 *vs*. negative control. ^#^*p* < 0.05 *vs*. KRN633 50 μM treated cells.

We evaluated the effects of VEGF/VEGFR2 signaling on tumor tropic ability of stem cells under the assumption that reduction of VEGF and VEGFR2 interaction would be correlated with the migratory ability of stem cells. KRN633 applied at 50 or 100 μM as a VEGFR2 inhibitor was used to treat 6-well cultured stem cells, and total RNA was extracted with RNA extraction solution after 3, 6, 9, or 24 h. The VEGFR2 gene was continuously expressed in stem cells following KRN633 treatment (Figure 4A), while there was no change in the expression level of the VEGFR2 gene (Figure 4B).

For analysis at the protein level, we harvested the total protein within one hour of KRN633 treatment. We then examined p-Erk1/2, p-Akt1/2/3, and c-fos protein expression by immunoblotting in HB1.F3.CD cells (Figure 4C). Interestingly, Erk1/2 phosphorylation was induced in HB1.F3.CD cells, and expression of p-Erk1/2 was more sustained in KRN633 100 μM treated stem cells for one hour when compared to control or 50 μM treated stem cells (Figure 4D and 4E). KRN633 treatment temporarily down-regulated p-Akt protein in the stem cells in a dose-dependent manner (Figure 4F), while the level of c-fos protein was not altered significantly (data not shown).

### Upregulation of BAX gene in response to 5-FU treatment

**Figure 5:**
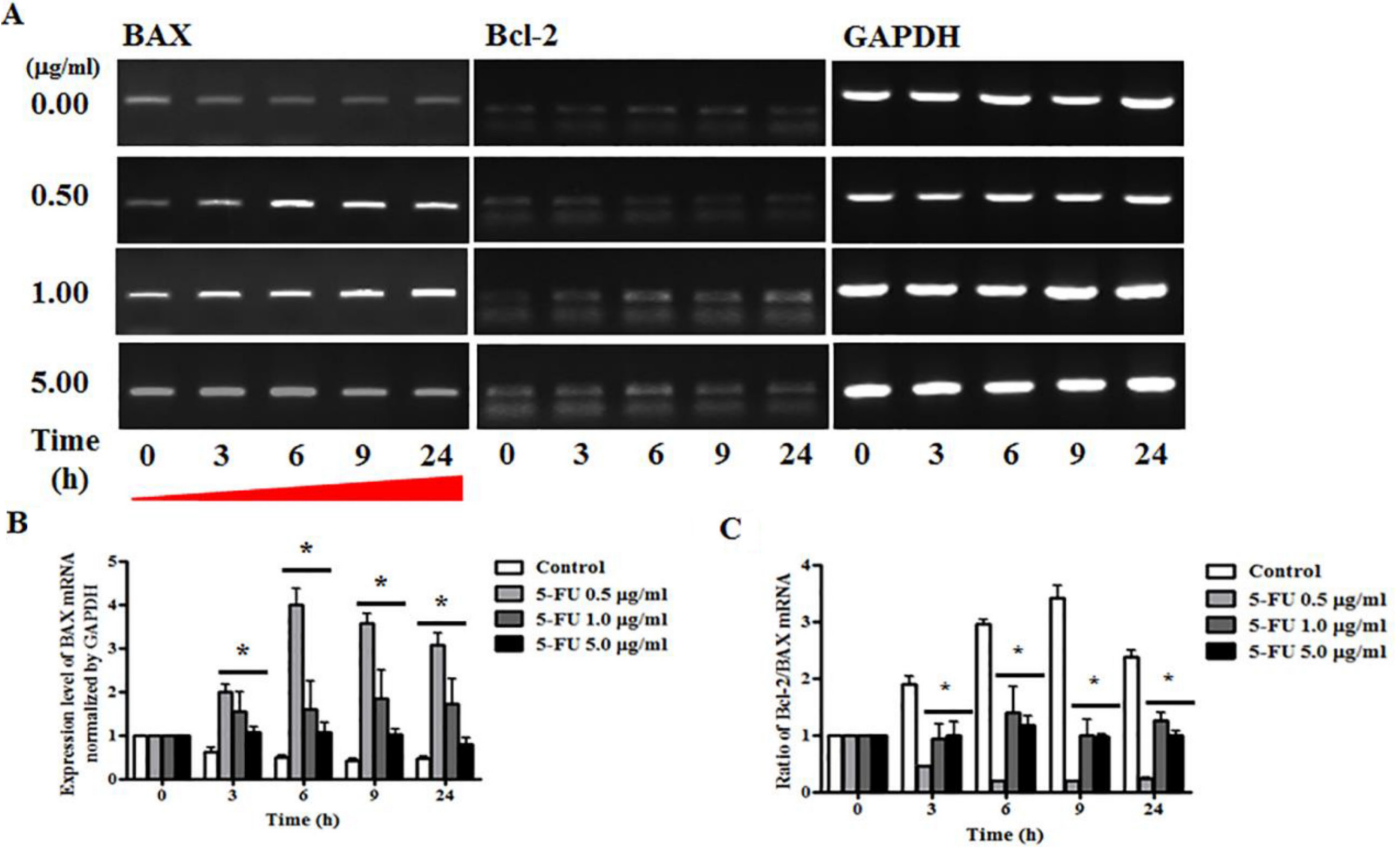
Effect of 5-fluorouracil (5-FU) on expression or apoptosis associated marker in MDA-MB-231 cells MDA-MB-231 cells were treated with 5-fluorocytosine (5-FC) at 5.0 μg/ml or 5-FU at 0.5, 1.0, and 5.0 μg/ml for 24 hours, after which BAX and Bcl-2 expression were determined by reverse transcriptase polymerase chain reaction (RT-PCR) analysis. GAPDH was used as a loading control and each sample was normalized to the GAPDH mRNA content. (**A**) BAX and Bcl-2 RNA expression levels. (**B**) Graph of BAX gene levels. (**C**) Graph of Bcl-2/BAX ratio. Data shown are the mean ± SD of three different experiments performed in triplicate. *p < 0.05 *vs*. negative control (no treatment with 5-FU).

To determine the mechanism underlying 5-FU-induced alteration of BAX and Bcl-2 genes, we conducted RT-PCR analysis of breast cancer cells after serial diluted 5-FU treatment (0.5, 1.0, and 5.0 μg/ml), and total RNA was extracted at 3, 6, 9, and 24 h. The results indicated that the apoptosis pathway was inhibited by regulation of the expression of BAX and Bcl-2 genes (Figure 5A). Specifically, these experiments showed that expression of the BAX gene increased significantly when treated with 5-FU of 0.5, 1.0, and 5.0 μg/ml, and this persisted until 24 h (Figure 5B). The ratio of the anti-apoptotic gene/pro-apoptotic gene was measured by semi-quantitative RT-PCR, and all 5-FU treated cells showed low Bcl-2/BAX ratios due to induction of apoptosis in breast cancer cells (Figure 5C).

### Alteration of apoptosis and proliferation related protein level in response to 5-FU

**Figure 6:**
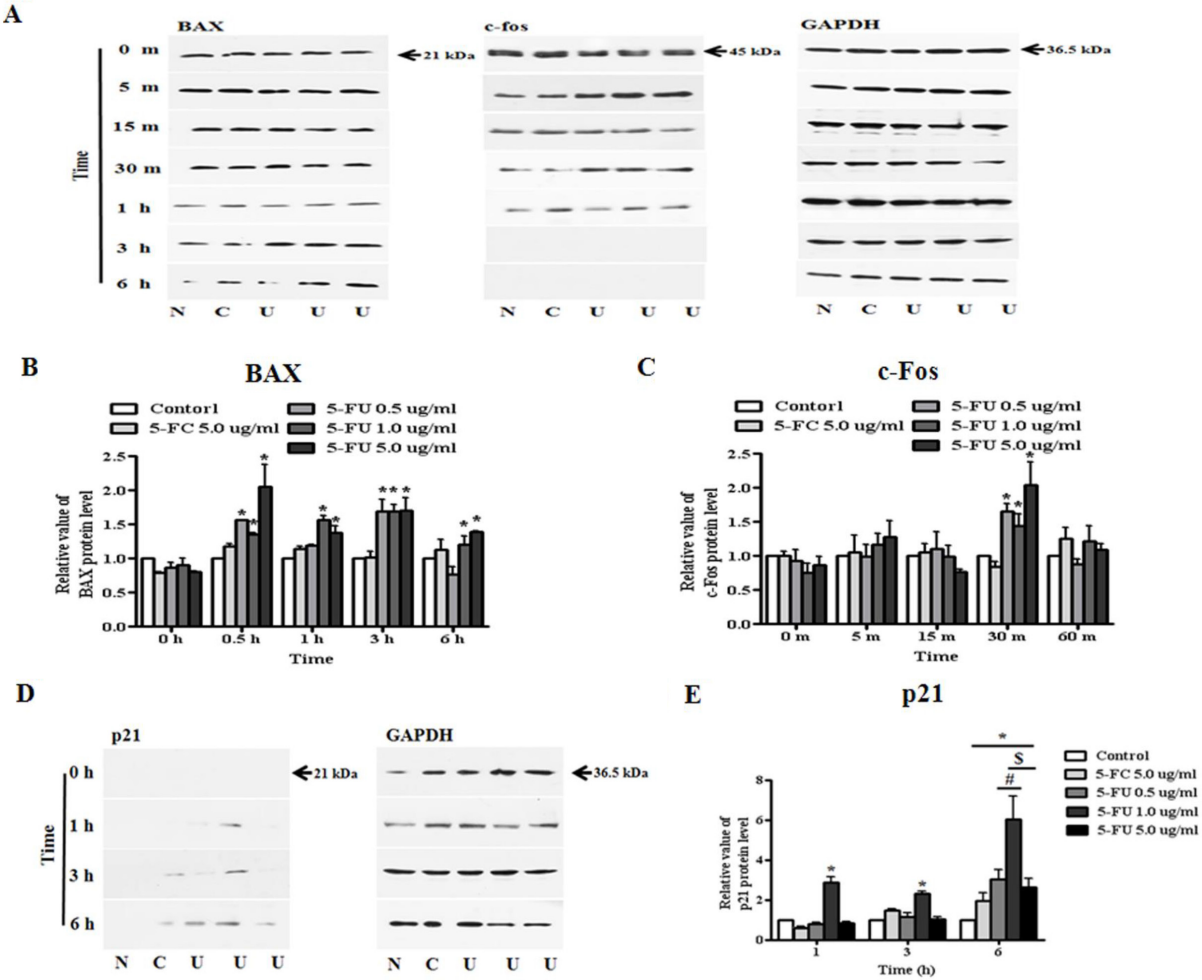
Alteration of apoptosis and proliferation related protein expression in MDA-MB-231 cells after 5-fluorouracil (5-FU) treatment To confirm the effects of 5-FU, serially diluted 5-FU (0.5, 1.0, and 5.0 μg/ml) and 5-fluorocytosine (5-FC) at 5.0 μg/ml were applied to MDA-MB-231 cells, which were then cultured in 6-well plates. Whole cell lysates were resolved by SDS-PAGE and immunoblotted with specific antibodies to BAX, c-fos, and p21 (Cip1/Waf1). (**A**) Expression of BAX and c-fos protein. (**B**) The relative value of BAX protein levels. **(C)** The relative value of c-fos protein levels. (**D**) The expression of p21 (Cip1/Waf1) protein. (**E**) Graph of p21 (Cip1/Waf1) protein levels. N: negative control (no treatment with 5-FU or 5-FC), C: 5-FC treatment (5.0 μg/ml), U: 5-FU treatment (0.5, 1.0, and 5.0 μg/ml, from the left). Data are presented as the mean ± SD of three different experiments each performed in triplicate. *p < 0.05 *vs*. negative control. #*p* < 0.05 *vs*. 5-FU 0.5 μg/ml treated cells. $*p* < 0.05 *vs*. 5-FU 5.0 μg/ml treated cells.

In this study, modulation of the activity of BAX, p21 (Cip1/Waf1), and c-fos upon exposure to 5-FU at 0.5, 1.0, and 5.0 μg/ml were evaluated. Proteins from untreated and 5-FC or 5-FU treated breast cancer cells were extracted in the presence of protease and phosphatase inhibitors. The data shown in Figure 6 indicate that the BAX protein was increased in 5-FU treated cancer cells from 30 min to 6 h (Figure 6A and 6B). Although almost no change in the expression of c-fos protein was observed, the expression level of the protein increased slightly after 5-FU treatment for 30 m (Figure 6C). Finally, we confirmed the expression of p21 (Cip1/Waf1) as a cyclin-dependent kinase inhibitor (Figure 6D). The 5-FU 1.0 μg/ml-treated group showed the first expression of the p21 (Cip1/Waf1) protein at 1 h after 5-FU treatment, while expression was observed in the 0.5 μg/ml-treated group at 3 h after treatment. Although this phenomenon lasted for six hours, p21 (Cip1/Waf1) protein was not observed in samples treated with 5.0 μg/ml 5-FU (Figure 6E).

### Association of other transcription factors

**Figure 7:**
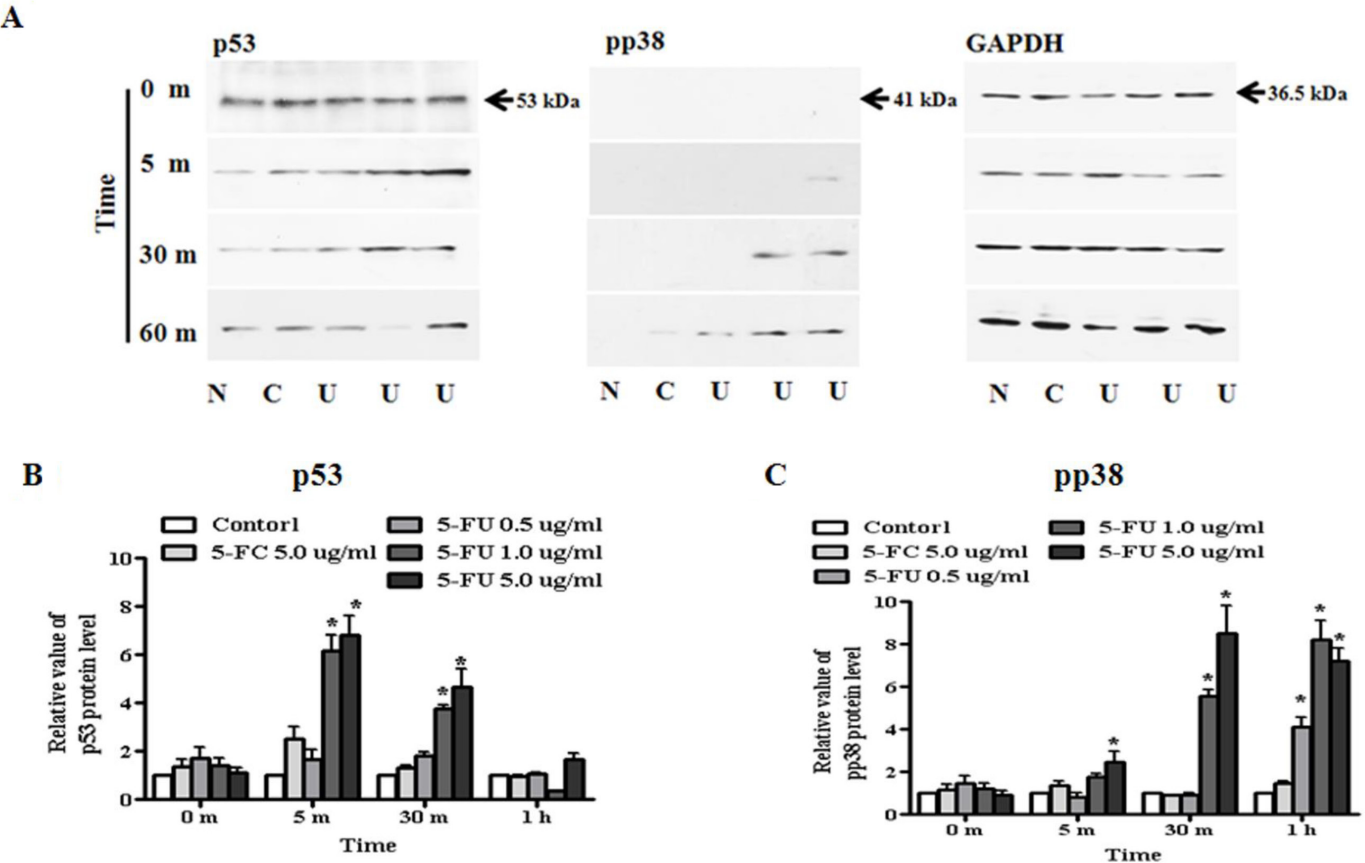
Regulation of transcriptional factor, p53 and pp38 in MDA-MB-231 cells MDA-MB-231 cells were treated with 5-fluorouracil (5-FU) in a dose-dependent manner for one hour. Whole cell lysates were separated by SDS-PAGE and immunoblotted with primary antibodies to p53, phospho-p38, and GAPDH. Each sample was normalized to its GAPDH protein. (**A**) Expression of p53 and pp38 protein. (**B**) The value of p53 protein levels. (**C**) The value of pp38 protein levels. N: negative control (no treatment with 5-FU or 5-FC), C: 5-FC treatment (5.0 μg/ml), U: 5-FU treatment (0.5, 1.0, and 5.0 μg/ml, from the left). Data are presented as the mean ± SD of three different experiments each performed in triplicate. *p < 0.05 *vs.* negative control.

Based on alteration of apoptosis and proliferation associated protein following 5-FU treatment, we tested whether expression of the transcriptional factors, p38 and p53, in MDA-MB-23l/Luc cells might inhibit cell proliferation and reduce cell viability (Figure 7A). For protein analysis, breast cancer cells were seeded in 6-well plates, treated with serially diluted 5-FU (0.5, 1.0, and 5.0 μg/ml) and cultured for up to six hours. Protein samples were collected at the indicated time points and analyzed for upstream apoptosis or proliferation associated protein. First, we investigated p53 expression upstream of BAX and p21 (Cip1/Waf1) protein. We found that, when compared with 5.0 μg/ml of 5-FC or 0.5 μg/ml of 5-FU, expression of p53 was significantly higher in 1.0 μg/ml or 5.0 μg/ml 5-FU treated breast cancer cells (Figure 7B). Additionally, phosphorylation of p38 as a regulatory factor of p53 was activated after 5-FU treatment, and this occurred in a dose-dependent manner (Figure 7C). Expression of pp38 protein increased by 6- or 8.5-fold in response to treatment with 1.0 or 5.0 μg/ml 5-FU, respectively, and was sustained for 1 h.

### Synergistic effect of 5-FU and IFN-β in MDA-MB-231 breast cancer cells

**Figure 8:**
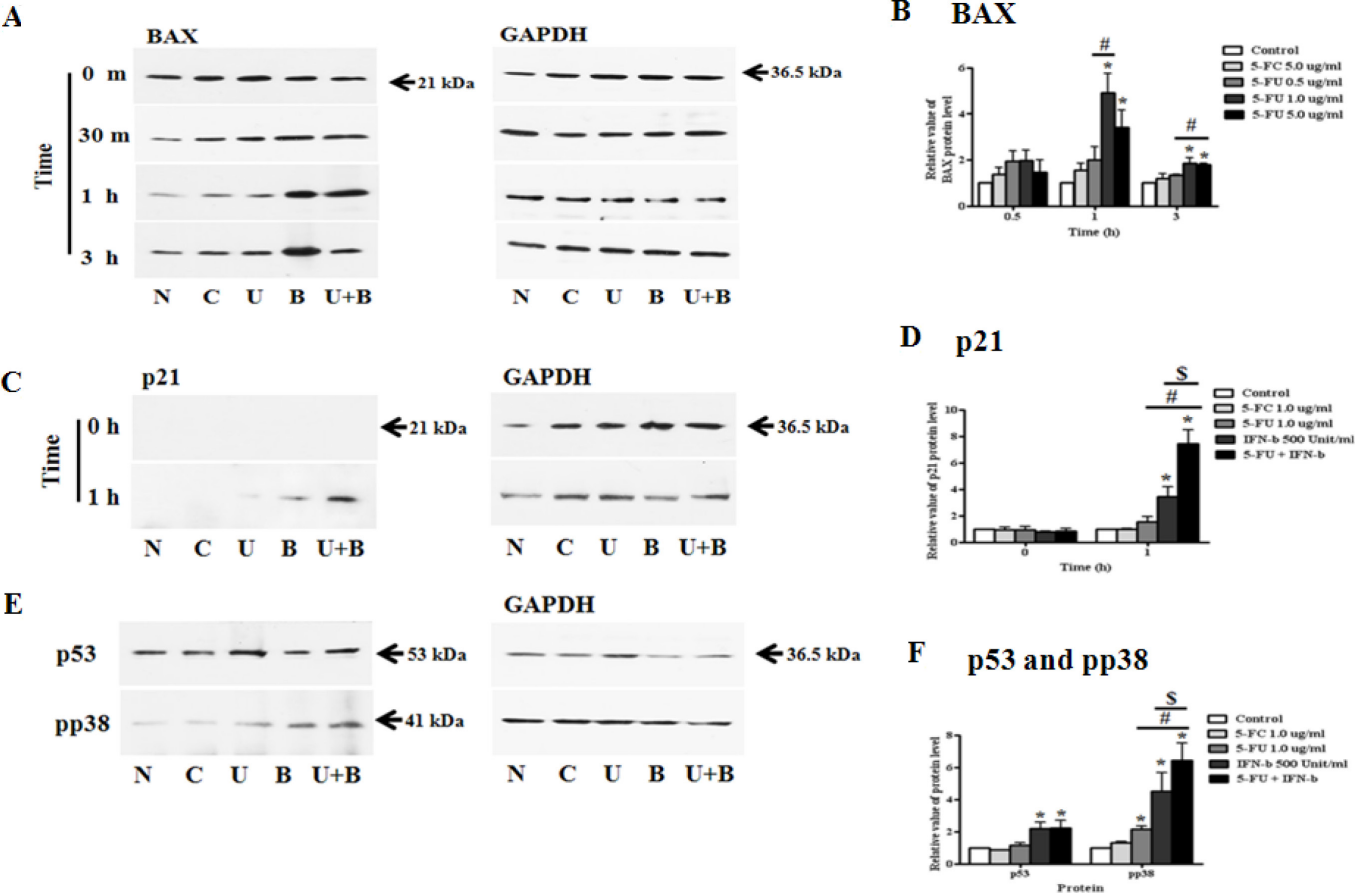
Synergistic effect of 5-fluorouracil (5-FU) and human interferon-beta (IFN-β) for treating MDA-MB-231 cells MDA-MB-231 cells were co-treated with 5-FU 1.0 μg/ml and IFN-β 500 Unit/ml for different lengths of time. Cell lysates were used for immunoblotting analysis and incubated with primary antibodies, BAX, p21 (Cip1/Waf1), p53, and phospho-p38. (**A**) Expression of BAX protein. (**B**) The relative value of BAX protein. (**C**) Expression of p21 (Cip1/Waf1) protein. (**D**) The relative value of p21 (Cip1/Waf1) protein. (**E**) Expression of p53 and pp38 protein. (**F**) The relative value of p53 and pp38 protein. N: negative control (no treatment with 5-FU or 5-FC), C: 5-FC treatment (1.0 μg/ml), U: 5-FU treatment (1.0 μg/ml), B: IFN-β treatment (500 Unit/ml), U + B (5-FU 1.0 μg/ml and IFN-β 500 Unit/ml co-treatment). Data are presented as the mean ± SD of three different experiments each performed in triplicate. *p < 0.05 *vs*. negative control. #*p* < 0.05 vs. 5-FU 0.5 μg/ml treated cells. $*p* < 0.05 *vs*. 5-FU 5.0 μg/ml treated cells.

In the next set of experiments, we investigated 5-FU and IFN-β in drug combination studies. Through various experiments, we determined the appropriate concentration of 5-FU for co-treatment. Samples were then co-treated with 5-FU 1.0 μg/ml and 500 Unit/ml IFN-β, after which each protein was harvested and analyzed by western blot analysis. BAX protein was strongly activated by IFN-β treatment when compared to 5-FU anti-drug treated cells, but a synergistic effect of cytokine and anti-cancer drug was observed in the co-treatment group (Figure 8A and 8B). Additionally, treatment with 5-FU and IFN-β led to a strong increase in p21 (Cip1/Waf1) expression relative to untreated or single treated (5-FC, 5-FU, and IFN-β) cells (Figure 8C and 8D). As expected, co-treatment with 5-FU and IFN-β rapidly induced the expression of pro-apoptotic protein or proliferation regulator in MDA-MB-231 cells. In addition to the increase in BAX and p21 protein, we investigated whether transcription factor p38 and p53 could be more phosphorylated in response to 5-FU and IFN-β co-treatment (Figure 8E). In the case of p53, the expression of protein increased in response to co-treatment with 5-FU and IFN-β for 15 min relative to other treatment cells (Figure 8F). We also performed immunoblot analysis to evaluate the phosphorylation levels of p38 in cells pretreated with 5-FU and IFN-β. After co-exposure for 30 min, MDA-MB-231 cells exhibited higher induction of p38 phosphorylation, as well as of the downstream growth factor signaling molecules p53, BAX, and p21 than cells treated with only 5-FU or IFN-β.

## DISCUSSION

To confirm the importance of NSCs expressing CD and/or IFN-β genes in the metastatic breast cancer animal model, we used three types of GESTECs, HB1.F3 as a parental cells, HB1.F3.CD as a representative expressing one suicide gene, and HB1.F3.CD.IFN-β cells as a representative expressing a suicide gene and a cytokine. The three types of stem cells were injected into mouse brains following injection of MDA-MB-231/Luc cells. Upon therapeutic analysis, NSCs expression of CD+/−IFN-β significantly inhibited growth of the tumor burden, while the co-expressing stem cells treated group showed a stronger reduction of tumor burden than the group treated with CD expressing stem cells alone. Although measurement of tumor burden using a veterinary caliper revealed no significant difference, the synergistically therapeutic effect of CD and IFN-β was confirmed through bioluminescence imaging or analysis of survival rate in the mouse model. Therapeutic gene expressing stem cells treated mice showed a significant decrease in bioluminescence signal intensity when compared with control or parental stem cells treated mice. Histopathological analysis also showed a trend toward significance when compared with mice treated with single therapeutic gene expressing stem cells. Necrosis zones of cancer cells and apoptosis were more frequently observed in HB1.F3.CD.IFN-β with 5-FC treated mice than in those treated with HB1.F3.CD and 5-FC. These results also indicated that modified NSCs have a strong therapeutic potential for prolongation of life span.

The migration of NSCs toward breast cancer cells was reported in previous studies [13, 31]. In a mouse model of MDA-MB-435 cells induced metastatic breast cancer, PKH26-posotive NSCs injected into the contralateral region were found in the brain areas of the tumor-bearing hemisphere [32]. In the present study, we measured several chemoattractant factors secreted by breast cancer cells, uPA, SDF-1α, VEGF, MCP-1, and SCF. In our previous studies, parental NSCs and HB1.F3 cells were confirmed to contain several related-receptors including uPA-related receptor (uPAR), VEGFR2, and SCF-related receptor (c-Kit) [25]. Rosova *et al.* showed that mesenchymal stem cells (MSCs) cultured in hypoxia increased their migration rates relative to normoxic conditions [33]. Based on these results, we hypothesized that VEGF/VEGFR2 signaling will affect the migratory effects of NSCs via ligand-receptor response secreted by cancer cells and stem cells, respectively. In the central nervous system (CNS), VEGF stimulates the expansion of NSCs and neurogenesis in various animal models, resulting in improved learning ability [34]. Following inhibition of VEGFR2 receptor expression in stem cells, we observed tumor tropic effects of CM-DiI stained stem cells by transwell assay. Although no changes in the levels of VEGFR2 RNA were observed, several VEGF/VEGFR2 pathway-associated downstream transcriptional factors showed altered protein expression, including Erk1/2 in the mitogen activated protein kinase (MAPK) signaling pathway and Akt in the PI3K/Akt signaling pathway. These results confirmed that a) phosphorylation of Erk1/2 increased, b) phosphorylation of Akt decreased, and then increased.

In a previous study, application of SDF-1α as a chemoattractant of factor to induce tumor tropism of stem cells increased phosphorylation of Erk1/2 in a primary subventricular zone (SVZ) culture [35]. However, expression of p-Erk1/2 was induced following treatment with VEGFR2 inhibitor in our study. Phosphorylation of Erk1/2 is related to stimulation of stem cells differentiation as well as migratory ability [36]. Therefore, it is likely that inhibition of VEGF/VEGFR2 signaling facilitated the differentiation of NSCs in the present study. Our data also showed that protein analysis of the stem cells in media containing KRN633 temporarily decreased phosphorylation of Akt. Under hypoxia, expression of Akt protein was likely an important factor in improved survival and retention of cells in damaged tissues and faster functional improvement [33]. Therefore, we suggested that PI3K/Akt signaling might be reduced when the amount of VEGF is insufficient. In another study, stress status of NSCs appears to be required to maintain their self-renewal via negative regulation of the PI3K/Akt pathway and neurogenesis by maintaining adequate levels of PI3K signaling [37]. Treatment with VEGFR2 inhibitor can promote increased Akt phosphorylation via activation of the MAPK pathway at a later time. Taken together, although NSCs may sustain an undifferentiated state and migratory ability through VEGF secreted by themselves, stem cells may show greater migration to the site of injury or tumor region due to excess VEGF secretion by damaged cells or cancer cells. Even though the phenomenon of tumor-tropic effects in stem cells is not clear, regulation of the MAPK and PI3K/Akt pathway may play a crucial role in this process through interaction of chemoattractant factors and their receptors. In our previous study, tumor cells modulate Erk1/2 and Akt signaling and migration of stem cells by secreting VEGF [38]. This further increased tumor-selectivity of stem cell/prodrug co-therapy. Overall, these results indicate that NSCs expressing the therapeutic gene may be a powerful tool for treatment of primary lung cancer or metastasis of lung cancer to the brain.

To investigate whether BAX, p21 (Cip1/Waf1), c-fos, p38, and p53 proteins are activated after treatment with 5-FU in the absence or presence of IFN-β, we incubated MDA-MB-231 cells under each condition with different concentrations of 5-FU (0.5, 1.0, and 5.0 μg/ml). The typical apoptosis and proliferation markers, BAX and p21 (Cip1/Waf1) expression, were observed in 5-FU+/−IFN-β treated groups. The response to drugs occurred rapidly after 5-FU and IFN-β co-treatment relative to other groups. Apoptosis is triggered by the imbalance between pro-apoptotic and anti-apoptotic protein of the Bcl-2 family, which determines the mitochondrial response to apoptosis stimuli [39]. We found a decrease in the ratio of the Bcl-2/BAX gene after 5-FU treatment, suggesting the activation of pro-apoptosis function of 5-FU leading to cell death. Although the Bcl-2/BAX ratio for induction of apoptosis showed the greatest decrease in response to 0.5 μg/ml of 5-FU, we selected the concentration of 1.0 μg/ml 5-FU following western blot analysis for co-treatment with human IFN-β in breast cancer cells. In this investigation, 5-FU and IFN-β stimulated BAX overexpression for apoptosis in breast cancer cells via synergistic effects. Similarly, p21 (Cip1/Waf1) expression was significantly increased in the 5-FU/IFN-β co-treated group relative to the 5-FU or IFN-β treatment only. We then investigated the upstream consequences of these effects by evaluating modulation of critical signaling cascades linked to BAX and p21 mediated pathways. Modulation of the activity of two critical signaling factors, p53 and p38, was evaluated upon exposure to 5-FU+/−IFN-β. p53 and p38 were phosphorylated in 5-FU+/−IFN-β treated breast cancer cells, and combined inhibition of 5-FU with IFN-β demonstrated synergy against these cells. These data provided a mechanistic rationale for investigation of whether NSCs expressing CD.IFN-β showed a synergistic effect through activation of p53 and p38 protein. In a further study, immune response of human IFN-β should be proved to analyze the exact mechanism of its synergistic effect, because IFN-β is a strong cytokine which can induce anti-viral and anti-cancer effects [40].

In conclusion, these results in this study demonstrate the potential for use of NSCs as an effective delivery system in the presence of a prodrug to target metastasis of brain tumor via the VEGF/VEGFR2 pathway. Co-expression of CD with the IFN-β gene induced apoptosis and suppressed cell proliferation via modulation of key signaling molecules such as p53 and p38. These findings suggest the possibility of developing new stem-cell based therapy for treating metastatic breast cancer.

## MATERIALS AND METHODS

### Cell culture

The breast cancer cell line, MDA-MB-231/Luc, which stably expresses firefly luciferase as a bioluminescent reporter gene, was purchased from KOMA biotechnology (Seoul, Korea). These cells were cultured in Dulbecco's modified eagle's medium (DMEM; Hyclone Laboratories, Inc., Logan, UT, USA) supplemented with 10% (v/v) heat inactivated fetal bovine serum (FBS; Hyclone Laboratory Inc.), 100 Unit/ml penicillin, 100 μg/ml streptomycin (Cellgro Mediatech Inc., Manassas, VA, USA), and 10 mM HEPES (Gibco, Carlsbad, CA, USA) at 37°C in a humidified 5% CO_2_ atmosphere. Human NSCs, HB1.F3, HB1.F3.CD and HB1.F3.CD.IFN-β were obtained from Chungang University (Seoul, Korea) and incubated in DMEM supplemented with 10% FBS, 100 Unit/ml penicillin, 100 μg/ml streptomycin, 10 mM HEPES and 0.1% antimycoplasmal agents (Invivogen, San Diego, CA, USA) at 37°C in a humidified 5% CO_2_ atmosphere. All cell lines were passaged using 0.05% trypsin/0.02% EDTA (Gibco).

### Xenograft and therapeutic effect of GESTECs

All animal experiments were approved by the Animal Care Committee of Chungbuk National University. Six weeks old female nude mice purchased from Central Laboratory Animal (Seoul, Korea) were housed in a pathogen free environment under a 12 h light/dark schedule with frequent ventilation. During the experiment, mice were provided with an autoclaved rodent diet (Central Lab. Animal Inc.) and water *ad libitum*. To establish metastatic breast cancer models in mice, MDA-MB-231/Luc cells (1 × 10^5^ cells/mice) were suspended in 8 μl of 1 × phosphate buffered saline (PBS) and directly implanted into the white matter of the right hemisphere [anterior/posterior (AP) +1.0 mm, medial/lateral (ML) +1.7 mm, dorsal/ventral (DV) −3.2 mm]. Two weeks later, 32 mice were randomly divided into four groups (each *n* = 8); 1) a negative control, 2) HB1.F3 cell treated group in the presence of 5-FC, 3) HB1.F3.CD cell treated group in the presence of 5-FC, 4) HB1.F3.CD.IFN-β cell treated group in the presence of 5-FC. The mice were injected into the left hemisphere with the stem cells (1 × 10^5^ cells/mice), HB1.F3, HB1.F3.CD, or HB1.F3.CD.IFN-β cells, pre-stained with 2 μM of chloromethylbenzamide-1,1′-dioctadecyl-3,3,3′-tetramethyl-indocarbocyanine perchlorate (CM-DiI; Invitrogen Life Technologies, San Diego, CA, USA). Control mice were injected with vehicle (8 μl PBS). After injection of stem cells and vehicle, 5-FC (500 mg/kg/day; Sigma-Aldrich Co., St. Louis, MO, USA) was administrated via an intraperitoneal (*i.p.*) injection (once a day, 5 days per week for 2 weeks). All mice were observed until the end point of the animal experiment, at which point the survival rate of stem cells treated mice was determined.

### Bioluminescence imaging

Mice in both groups were imaged weekly using an IVIS™ Spectrum Scanner (Xenogen; Caliper Life Sciences, Hopkinton, MA, USA) containing a charge-coupled (CCD) camera with photon emitting properties. Briefly, 150 μg/kg of luciferase substrate D-luciferin (Promega, Madison, WI, USA) in dissolved solution was injected *i.p.* into each mouse at 10 min prior to imaging, after which they were anesthetized with 1–3% isoflurane. The minutes later, bioluminescent tumor cells were detected by a CCD camera mounted in a light-tight box and tumor regions were quantified using a total photon counter (photon/s). Living Image controlled imaging analysis software (Caliper Life Sciences, Waltham, MA, USA). Regions-of-interest (ROI) of the same size and shape were used for all mice throughout the study.

### Hematoxylin and eosin staining (H & E staining)

Paraffin-fixed brains from control and NSCs treated mice were used to conduct histopathological analysis. The tumor tissues were collected after sacrificing the mice and then fixed in 10% normal formalin (Sigma-Aldrich Co.), embedded in paraffin blocks and cut with a sliding microtome (3 μm sections). After deparaffination and rehydration, the slides were stained using hematoxylin (Sigma-Aldrich Co.) and eosin (Sigma-Aldrich Co.). To prevent sample contamination, stained slides were hydrated and mounted using mounting solution. The size and location of the tumor mass in the brain was observed by light microscopy using a BX51 microscope (Olympus, Japan).

### Immunohistochemistry (IHC)

Paraffin embedded brain sections were subjected to IHC staining. Antigen retrieval of brain slides was performed by microwave for 10 min in a chamber containing citrate buffer (0.01 M; pH 6.0). Following antigen retrieval, tissue slides were placed in 0.3% methanol/hydrogen peroxidase (Sigma-Aldrich Co.) for 30 min to quench the endogenous peroxidase. To block the non-specific binding of antibodies, this slide was incubated with 10% normal goat serum (Vector Laboratories, Burlingame, CA, USA) for 1 h. Subsequently, the slides were incubated overnight with a mixture of anti-PCNA (1:100, Abcam plc., Cambridge, UK) primary antibody in 5% bovine serum albumin (Sigma-Aldrich Co.) in a humidified chamber. The next day, the slides were washed three times in 1 × PBS-T (pH 7.4) and then incubated with appropriate biotinylated secondary antibodies (Vector Laboratories) for 30 min at room temperature. Slides were subsequently rinsed with PBS-T for 10 min, after which Vectastain Universal Elite ABC kit reagent (Vector Laboratories) was applied for 30 min. Immunoreactive complexes were detected using DAB substrate (Sigma-Aldrich Co.) and counted after staining with hematoxylin. Finally, slides were mounted with a cover slip using mounting medium. All slides were visualized under a BX51 light microscope for digital photography.

### Transwell assay

To investigate the effects of VEGF/VEGFR2 signaling pathway on the migration of GESTECs, we performed an *in vitro* cell migration assay using a 24-well transwell with a pore size of 8 μm (BD Biosciences, Franklin Lakes, NJ, USA). MDA-MD-231/Luc cells (1 × 10^5^ cells/well) were cultured in the lower chamber of the transwell and fibronectin was applied to the transwell to induce the adhesion of migrated GESTECs. To inhibit VEGF/VEGFR2 signaling, stem cells was exposed to KRN633 (Selleckchem, Houston, TX, USA) diluted media before being seeded in the transwell. Briefly, after starvation in serum free media, 100 μM KRN633 was applied to each stem cell for 1 h. The CM-DiI pre-stained GESTECs (1 × 10^5^ cells/well) were then seeded in the upper chamber of transwell after treatment with KRN633. The chamber was subsequently incubated at 37°C for 24 h. After incubation, the non-migrated cells were scraped with a plastic blade and then fixed in cold methanol. The upper chambers of transwell were washed with PBS and stained with 200 ng/ml 4′, 6-Diamidino-2-phenylindole (DAPI; Sigma-Aldrich Co.). Finally, migrated cells were examined with an Olympus microscope (IX71 Inverted Microscope, Olympus, Japan) connected to a fluorescence detector.

### Real-time and reverse transcription (RT)-PCR

Total RNA of breast cancer cells and stem cells was extracted with TRIzol reagent (Invitrogen Life Technologies) RNA extraction solution according to the manufacturer's recommendation. Reverse transcription reaction was performed using 1 μg total RNA with murine leukemia virus reverse transcriptase (MMLV-RT; iNtRON Biotechnology, Sungnam, Kyeonggido, Korea), 10 pM dNTP (Bioneer, Deajeon, Korea), nonamer random primer (TaKaRa Bio., Shiga, Japan), 5 × RT buffer (iNtRON Biotechnology) and RNase inhibitor (iNtRON Biotechnology).

The real time PCR mixture was composed of 2 × SYBR green premix (TaKaRa Bio.), ROX (TaKaRa Bio.) as a reference dye, and sense and antisense primers (Bioneer). PCR was conducted to detect chemoattractant factor genes including urokinase-type plasminogen activator (uPA), SDF-1α, VEGF, monocyte chemotactic protein 1 (MCP-1), and stem cell factor (SCF). The PCR program consisted of 40 cycles of denaturation at 95°C for 15 s, annealing at 58°C for 20 s, and extension at 72°C for 15 s, and all analyses were carried out in triplicate for each sample. Glyceraldehyde 3-phosphate dehydrogenase (GAPDH) was used for normalization. The mRNA levels of these genes were determined using the 2^−ΔΔCt^ method and the specific primer sets listed in Table 1.

**Table 1:** Sequences of specific sense/antisense primer sets

mRNA		Sequence (5′→3′)
uPA	Sense	GGCAGGCAGATGGTCTGTAT
	Antisense	TTGCTCACCACAACGACATT
SDF-1α	Sense	GTGTCACTGGCGACACGTAG
	Antisense	TCCCATCCCACAGAGAGAAG
MCP-1	Sense	CAAGCAGAAGTGGGTTCAGGA
	Antisense	TCTTCGGAGTTTGGGTTTGC
VEGF	Sense	CCAGCACATAGGAGAGATGAGCTT
	Antisense	TCTTTCTTTGGTCTGCATTCACAT
SCF	Sense	GGCAAATCTTCCAAAAGACTACA
	Antisense	GCCTTCAGAAATATTTGAAAACTTG
GAPDH	Sense	ATGTTCGTCATGGGTGTGAACCA
	Antisense	TGGCAGGTTTTTCTAGACGGCAG

To analyze the mRNA levels of VEGFR2 or BAX/Bcl-2 genes in the MDA-BD-231 cells following KRN633 or 5-FU (Sigma-Aldrich Co.) treatment, PCR was performed using cDNA template, Taq polymerase (iNtRON Biotechnology), dNTP, 10 × PCR buffer (iNtRON Biotechnology), and specific primer sets (Bioneer). The PCR program consisted of 30 cycles of denaturation at 95°C for 30 s, annealing at 58°C for 30 s, and extension at 72°C for 30 s. PCR products were separated by electrophoresis on 1.5% agarose gel containing ethidium bromide (EtBr; Sigma-Aldrich Co.) and analyzed using Gel Doc 2000 (BioRad Laboratories Inc., Hercules, CA, USA). GAPDH was used as an endogenous control for normalization.

### Western blot

Following 5-FU and/or IFN-β treatment of breast cancer cells and KRN633 treatment of stem cells, whole cell lysates were extracted from the cancer and stem cells. Briefly, samples were placed in protein extraction solution (1 × RIPA solution; 50 mM Tris-HCl, pH 8.0, 150 mM NaCl, 1% NP-40, 0.5% deoxycholic acid, and 0.1% sodium dodecyl sulfate) with protease and phosphatase inhibitor cocktail (Roche Applied Science, Mannheim, Germany), incubated overnight at 4°C, and then centrifuged at 14,000 rpm for 30 min. The concentration of the protein was then determined using bicinchoninic acid (BCA; Sigma-Aldrich Co.) and copper (II) sulfate (Sigma-Aldrich Co.) mixture. Next, 40 μg of whole cell lysate was resolved by 12% SDS-polyacrylamide gel electrophoresis (SDS-PAGE), after which fractionated proteins were transferred to polyvinylidene difluoride (PVDF) transfer membrane (BioRad Laboratories Inc.). The membrane was subsequently blocked with 5% skim milk (BioRad Laboratories Inc.) blocking buffer to inhibit the non-specific interaction with primary antibody for 2 h and then washed four times in 1 × TBS buffer (adjusted to pH 7.6 with HCl) containing 0.1% (v/v) Tween 20 (BioRad Laboratories Inc.). Next, the membrane was incubated with primary antibody, mouse monoclonal anti-p21 (Cip1/Waf1) (1:1,000 dilution, Cell Signaling Technology, Inc., Danvers, MA, USA), anti-p53 (1:1,000 dilution, Santa Cruz Biotechnology, Inc. Dallas, TX, USA), anti-BAX (1:1,000 dilution, Cell Signaling Technology, Inc.), anti-GAPDH (1:1,000 dilution, Santa Cruz Biotechnology, Inc.), rabbit polyclonal anti-phospho-Erk1/2 (1:1,000 dilution, Cell Signaling Technology, Inc.), anti-phospho-Akt1/2/3 (1:1,000 dilution, Cell Signaling Technology, Inc.), anti-c-fos (1:2,000 dilution, Abcam plc., Cambridge, UK), or anti-phospho-p38 (1:1,000 dilution, Cell Signaling Technology, Inc.) in 1 × TBS with 3% (w/v) BSA (Sigma-Aldrich Co.) and 0.1% (v/v) Tween 20 overnight at 4°C. Samples were then incubated with specific antibody followed by the appropriate horseradish peroxidase (HRP) conjugated secondary antibody, goat anti-mouse IgG (1:3,000 dilution, Bio-Rad Laboratories Inc.) or goat anti-rabbit IgG (1:3,000 dilution, Santa Cruz Biotechnology, Inc.) for 2 h. Positive immunoreactive proteins were detected using an ECL West-Q Chemiluminescent Substrate Plus Kit (GenDEPOT, Barker, TX, USA).

### 5-FU and IFN-β treatment

For analysis of 5-FU effects in stem cell therapy, cancer cells were treated with various concentrations of 5-FU (0.5, 1.0, and 5.0 μg/ml), after which proteins were harvested at 5 m, 10 m, 15 m, 30 min, 1 h, 3 h, and 6 h following 5-FU treatment. To measure the expression of the apoptotic genes BAX and Bcl-2, MDA-MB-231 cells were exposed to 0.5, 1.0, and 5.0 μg/ml of 5-FU for 3, 6, 9, and 24 h. To investigate the synergistic effects of 5-FU and IFN-β (PBL Assay Science, Piscataway, NJ, USA), we co-treated samples with 1.0 μg/ml of 5-FU and 500 Unit/ml of human IFN-β in cultured MDA-MD-231 cells. Finally, whole protein was extracted using protein extraction solution.

### Statistical analysis

Experiments for RNA or protein quantification were carried out three times, after which statistical analyses were conducted using Graph Pad Prism 5 (Graph Pad Software, San Diego, CA, USA). Significant differences among groups were identified by one-way ANOVA followed by Tukey's test. Probability values < 0.05 were considered significantly different.
